# Sudden unexpected death in epilepsy (SUDEP): Risk management of pediatric patients with epilepsy

**DOI:** 10.1002/epi4.70214

**Published:** 2026-01-20

**Authors:** Laura Lutz, Lena Luise Becker, Alwina Koch, Angela M. Kaindl

**Affiliations:** ^1^ Department of Pediatric Neurology Charité—Universitätsmedizin Berlin Berlin Germany; ^2^ Center for Chronically Sick Children Charité—Universitätsmedizin Berlin Berlin Germany; ^3^ German Epilepsy Center for Children and Adolescents Charité—Universitätsmedizin Berlin Berlin Germany; ^4^ Charité Pediatric Head and Neck Center Charité—Universitätsmedizin Berlin Berlin Germany; ^5^ German Center for Child and Adolescent Health (DZKJ) Partner Site Berlin Germany; ^6^ Institute of Cell and Neurobiology Charité—Universitätsmedizin Berlin Berlin Germany

**Keywords:** DRE, drug‐resistant epilepsy, epilepsy, seizure‐detecting device, SUDEP

## Abstract

**Objective:**

Sudden unexpected death in epilepsy (SUDEP) is the leading cause of death in people with epilepsy with an incidence of 1:1000. The primary risk factors for SUDEP are generalized or focal to bilateral tonic–clonic seizures. Preventive measures like nighttime monitoring devices and resuscitation training address modifiable risk factors. Nevertheless, SUDEP awareness remains insufficient. This study aimed to analyze risk awareness and behavior and to evaluate the impact of a questionnaire.

**Methods:**

In a monocentric cross‐sectional pediatric study, routinely used questionnaires were analyzed retrospectively regarding SUDEP awareness rates and monitor ownership.

**Results:**

A total of 498 patient families completed the questionnaire between December 2023 and July 2024. At the time of the survey, 58% recalled having been informed about SUDEP before the questionnaire. The SUDEP questionnaire led to an education in the following outpatient clinic in 25%. A further 9% had been counseled within the following 6 months, leading to an education rate of 92%. Monitoring devices were used in 46% of patients, with nocturnal monitoring being most frequently used in children <4 years. Among those who did not use a monitor and provided reasons against monitoring, half had SUDEP risk factors. Additionally, one third of the responders reported not using their seizure detecting device, with false alarms (28%) being the most frequently cited reason.

**Significance:**

All patients with epilepsy should be counseled about epilepsy‐related risks and preventive measures. Our study highlights SUDEP education gaps and the need for targeted counseling strategies to contribute to SUDEP prevention. The findings show that a structured questionnaire can effectively identify education gaps, enhance counseling efforts, and improve health literacy. Furthermore, there is a need for the development of novel, reliable seizure detection devices.

**Plain Language Summary:**

Sudden unexpected death in epilepsy (SUDEP) remains the most common cause of death in people with epilepsy. That is why it is important to take steps to prevent it. We still do not fully understand why SUDEP occurs and how it can be completely prevented. But it is very important to be aware of the risk. This enables those affected and their families to take measures to reduce the danger. This text explains how to inform more people about SUDEP. It also shows a possible role of nighttime monitoring devices in the prevention of SUDEP.

**Key points**

- Sudden unexpected death in epilepsy (SUDEP) is the leading cause of death in people with epilepsy. This study aimed to analyze SUDEP risk awareness and risk behavior.
- The routine use of a standardized SUDEP questionnaire increased the education rate by 25%.
- An incorrect risk assessment can lead to inadequate risk behavior. Among those who did not use a monitor, half had SUDEP risk factors not recognized by their caregivers.
- Nocturnal monitoring was most frequently used in children <4 years.
- Existing seizure detection devices were not in use mostly because of false alarms.

## 1 INTRODUCTION

Epilepsy is one of the most common neurologic disorders worldwide and is associated with a reduced life expectancy.^1^, ^2^, ^3^, ^4^, ^5^, ^6^ While an increased risk of injury or drowning during a seizure, suicide, cardiac arrhythmia or sudden cardiac death, and life‐threatening status epilepticus^4^, ^7^, ^8^ contributes to the mortality, the leading cause of death is sudden unexpected death in epilepsy (SUDEP).

SUDEP is defined as a sudden non‐traumatic and non‐drowning death in patients with epilepsy, with or without evidence of a seizure, in which no other cause of death than epilepsy itself can be identified.^9^ The incidence of 1 in 1000 epilepsy patients per year is similar in children and adults.^10^, ^11^ The exact mechanism underlying SUDEP remains unclear, although postictal brainstem suppression with consecutive hypoventilation, hypoxia, and subsequent asystole has been reported in some individuals.^7^ Generalized tonic–clonic seizures (GTCS) and focal to bilateral tonic–clonic seizures are the main risk factors of SUDEP.^7^, ^12^, ^13^, ^14^, ^15^, ^16^ Additional major risk factors include lack of seizure freedom, drug‐resistant epilepsy (DRE), nocturnal seizures, being unobserved or living alone, and the presence of specific epilepsy syndromes such as developmental and epileptic encephalopathies (DEE), including Dravet syndrome. SUDEP has also been reported in patients with benign self‐limiting epilepsies (e.g., SeLECTs).^17^, ^18^, ^19^, ^20^, ^21^

People with epilepsy are three times more likely to die from sudden cardiac death than the general population.^8^ Cardiologic assessments and management may be important given the data indicating a cardiac component in SUDEP victims.^22^ An additional SUDEP risk is suspected due to seizure‐induced myocardial ischemia, cardiac dysfunction, and secondary cardiac arrhythmia.^23^ Long‐term electrocardiogram (ECG) heart rate variability can be an important parameter to further assess the risk of SUDEP.^24^, ^25^

There is no standardized guideline on SUDEP prevention and patient education. Knowledge of SUDEP is crucial for the implementation of effective preventive strategies. Timely detection of a potential nocturnal cardiac arrest, for example by nighttime monitoring, is essential to alert parents and caregivers. Only if they are adequately informed and prepared can appropriate resuscitation measures be promptly initiated. While current evidence supporting the effectiveness of nighttime monitoring devices in reducing SUDEP risk is limited,^26^ the hypothesis remains plausible,^27^, ^28^ particularly given the ethical challenges associated with conducting randomized controlled trials in this context.

Given the great public health success of campaigns focusing on the sudden infant death syndrome (SIDS) in the 20th century, it is assumed that the SUDEP risk can be reduced through appropriate education and awareness measures, as well as approaches such as seizure control, seizure detection, and the rapid initiation of basic life support.^15^, ^19^

**FIGURE 1:**
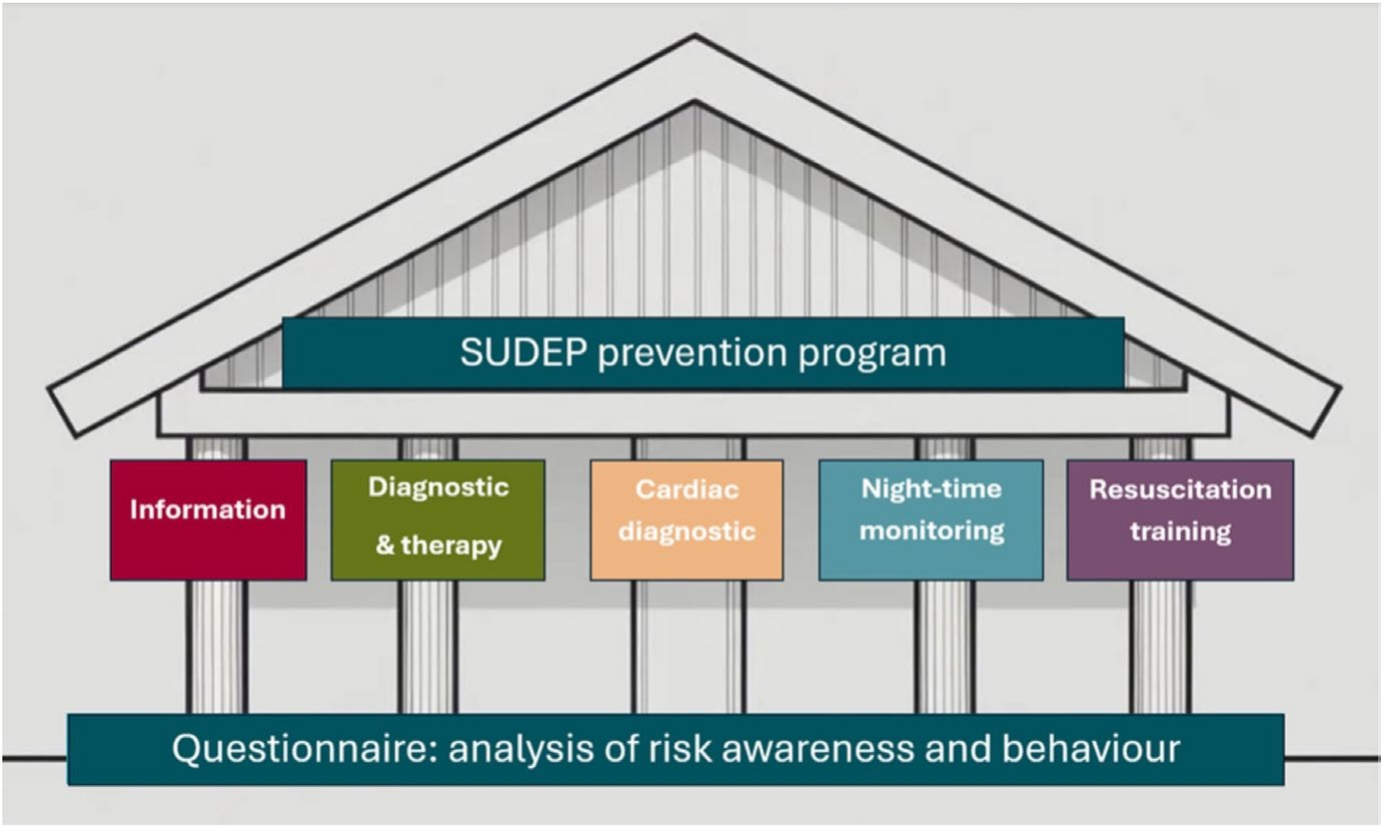
The five pillars of the SUDEP prevention program at Charité.

To address the lack of knowledge about SUDEP among patients, we developed the SUDEP Prevention Program to improve patient outcomes and reduce mortality by providing comprehensive information about epilepsy and its risks, as well as equipping patients with the skills to implement preventive measures and make informed treatment decisions. The program included the provision of detailed information and guidance on epilepsy diagnostics, treatment options, and associated risks through information packages and seminars for patients, guardians, and teachers. The information package contained a questionnaire on SUDEP. Also, individual risk assessment, coaching, and support for selecting and adjusting suitable monitoring systems, as well as assistance with insurance applications, were provided. Resuscitation training specifically designed for pediatric resuscitation was also offered. Furthermore, we introduced documentation of SUDEP education status and monitor ownership in the diagnosis lists included in the medical letters sent to the primary pediatricians and patient families. This ensured standardized reporting and information dissemination (see Figure 1).

In this study, we evaluated SUDEP associated risks using a questionnaire at the German Epilepsy Center for Children and Adolescents at Charité. The aim of the study was to identify gaps in information, obstacles, and reservations regarding appropriate risk management to address these more effectively in the future.

## 2 PATIENTS AND METHODS

### 2.1 Study design and participants

The present study was a monocentric, cross‐sectional pediatric study conducted on the patient population of the German Epilepsy Center for Children and Adolescents at Charité—Universitätsmedizin Berlin, Germany. The study was approved by the local ethics committee (approval no. EA2/112/24).

SUDEP questionnaires, used routinely in the framework of consultations, were analyzed retrospectively to assess SUDEP awareness rates and monitor ownership, CPR training, cardiologic diagnostics, co‐sleeping. The development of the questionnaire was based on observations from everyday clinical practice. The questions were compiled specifically to assess the current situation and for internal quality control purposes. The items were formulated independently. The final version comprised 25 items, 13 of which could be answered dichotomously (yes/no); the remaining questions provided answer options; multiple answers and own answers were possible. Experts in the field of psychology evaluated the items in terms of relevance, comprehensibility, and completeness. Overall, the psychologists confirmed the suitability of the content for collecting the desired data. The questionnaire is primarily a tool for retrospective status assessment with predominantly dichotomous (yes/no) answer formats; a pilot study and a pretest were not conducted. Compared to the SUDEP and Seizure Safety Checklist,^29^ which has been used in adult patients in the United Kingdom since 2015 and in pediatric patients since August 2024, this questionnaire also aimed to ask about previous cardiologic diagnostics and resuscitation training, as well as to record the type of monitoring systems used and the reasons for or against nighttime monitoring.

Pediatric patients aged 0–18 years who were treated for seizures at the German Epilepsy Center for Children and Adolescents and who completed the questionnaire (with the help of their parents/guardians) were included. A total of 500 patients with epilepsy or their parents/guardians answered the questionnaire. Data from adult patients were not included.

Three different age groups were distinguished: kindergarten (0–6), primary school (7–12 years), and secondary school‐aged children (13–18 years). In addition, the subgroup of children under 4 years of age was assessed separately, given that the most popular nighttime monitoring device in this study was not approved for this age group.

### 2.2 Intervention

The paper questionnaire was handed out to patients and their families as part of routine care over a 7‐month period. Questions included seizure types and occurrence/frequency, resuscitation training, co‐sleeping, and nighttime monitoring. Respondents were asked when and where they had been informed about SUDEP, whether they were aware of the possibility of resuscitation training and nocturnal monitoring, and whether co‐sleeping was practiced. They were also asked whether a monitoring device was being used, and if so, which one. Nocturnal monitoring systems included pulse oximeters measuring heart rate and oxygen saturation, as well as Nightwatch®, which detected abnormal limb movements and heart rate alterations through an algorithm.^30^ Other monitoring devices included Epicare 3000®, Epicare free®, and Epicare mobile®.^31^ Participants were also asked whether the prescribed monitor was being used, whether an additional monitor had been requested, whether any issues had arisen during the insurance request process, and to give reasons for use or non‐use of monitors. Additionally, the questionnaire assessed whether cardiologic diagnostics had been carried out. The detailed questionnaire can be found in Figure S1. In this study, we retrospectively analyzed all questionnaires in the period 12/2023 to 07/2024.

For younger patients and older patients with developmental delay, the parents/guardians filled out the questionnaire; older patients with normal cognition starting around the age of 10 years filled out the questionnaire independently or together with their parents/guardians. The questionnaires were filled out prior to outpatient consultations or after admission as inpatients, analyzed by an epilepsy specialty nurse and the attending physician, and used for subsequent individual counseling of the families. In the event of a lack of knowledge about SUDEP, the education was carried out immediately in the following outpatient clinic, if possible. Thus, the questionnaire served both: first, to document the current situation, and second to identify the knowledge gap by the epilepsy specialty nurse or the attending physician and then to intervene and educate the family. The detection of information and treatment gaps enabled the immediate initiation of educational and treatment measures, such as oral explanation, providing additional information material, adjusting epilepsy treatment (e.g., modifying anti‐seizure medication [ASM] or initiating other treatment options and providing a monitor application, etc.). The information from the questionnaires was then retrospectively combined with clinical data obtained from medical files such as diagnosis, seizure type(s), seizure frequency, type of epilepsy or epilepsy syndrome and ASM. All data were subsequently pseudonymized and entered into an electronic, RedCap‐based epilepsy database developed at our institution (EpiBASE©).

### 2.3 Statistics

Data analysis was conducted using descriptive statistics. Statistical analysis was performed with R version 4.4.2 (https://cran.r‐project.org/bin/windows/base/) using the package *ggplot2* and *lubridate*. In particular, the group of patients with higher SUDEP risk factors was examined, and their risk management strategies (preventive measures by means of resuscitation training, nighttime monitoring system) were analyzed.

## 3 RESULTS

Questionnaires were obtained from parents or guardians of 498 children with epilepsy aged 0–18 years (mean = 8.8 years) treated as inpatients or outpatients at our center (see Table 2). The median age at epilepsy diagnosis was 9 years, with 69% being diagnosed >1 year of age. The mean age of epilepsy onset was 4.4 years (range 0–16). There was an almost equal sex distribution of 50% female and 49% male, 1% diverse. Forty‐seven percent of the patients could be assigned to an epilepsy syndrome. Almost one third (28%, *n* = 140) of the patients were diagnosed with DEE; seven patients were diagnosed with Dravet syndrome. Developmental disorders were the most common comorbidities, affecting 54% (*n* = 267) of the cohort. A quarter (26%, *n* = 127) suffered from DRE.

### 3.1 Knowledge about SUDEP

**FIGURE 2:**
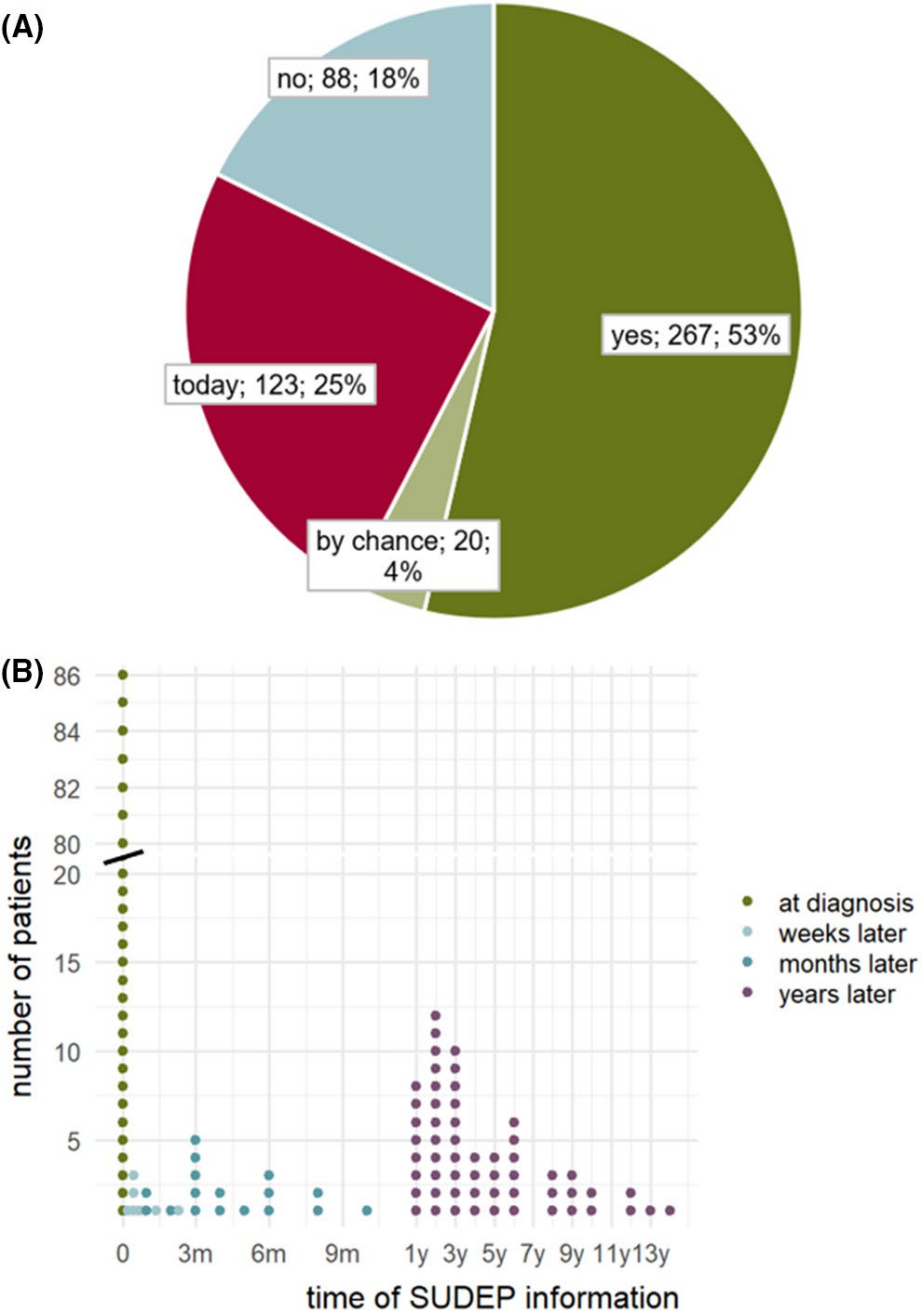
SUDEP education. (A) Are you informed about SUDEP? Frequency of SUDEP education. (B) Timing of SUDEP counseling. Two hundred and sixty‐seven patients stated that they had been informed: A third (32%, *n* = 86) reported having been informed about SUDEP at the time of diagnosis, while 45% (*n* = 119) reported having been informed about SUDEP at a later time: On average 39.4 months/3.3 years after diagnosis (months: Median 3.0, mean 3.974, minimum 1.0, maximum 10.0; years: Median 3.0, mean 4.579, minimum 1.0., maximum 14.0).

In the survey, 54% (*n* = 267) of patients and/or their parents or guardians indicated that they had already been informed about SUDEP by us (see Figure 2). 4% (*n* = 20) stated that they had heard about SUDEP by chance. Of the 211 patients (42%) who stated in the survey that they were unaware of SUDEP, a total of 123 patients (25%) could be directly informed about SUDEP through verbal explanations in the following visit. According to the doctor's letter, a further 9% of patient families were informed at a later time point. In total, the education rate was 92% in our center.

### 3.2 Nighttime monitoring systems

In total, 46% (*n* = 230) of patients indicated that they had a monitoring device, while 49% (*n* = 245) stated that they did not. Five percent (*n* = 23) did not provide any information. A total of 27% (*n* = 136) of patients stated that they were not aware of the possibility of monitoring. This was changed through counseling on the day of the interview in 76 cases; other cases were counseled later. German health insurance covers the costs of a nighttime monitoring system if there is a high risk of SUDEP. Still, 25% (*n* = 54) of all patients who answered this question reported experiencing problems with cost coverage. Notably, 37% (*n* = 20) of these patients were assigned to one of the high‐risk groups for SUDEP (for high‐risk groups for SUDEP, see Tables 1 and 2).

**TABLE 1:** SUDEP risk factors and preventive measures.

SUDEP risk factors	Preventive measures
Generalized tonic–clonic seizures and focal to bilateral tonic–clonic seizures (GTCS*) Nocturnal seizures Lack of nighttime monitoring/unobserved sleep Lack of seizure freedom Drug‐resistant epilepsy (DRE)	Precise diagnostics
	Therapy optimization with the aim of seizure freedom, avoiding GTCS*
	Detecting nocturnal seizures, education and advice on nighttime monitoring
	Resuscitation training for parents and caregivers
	Preparation of an individual emergency plan
Poor medicine adherence	Education and counseling about SUDEP, psychological and socio‐medical support
Cardiac factors	Cardiac diagnostics: ECG, echocardiography, long‐term ECG
Genetic disposition (e.g., Davet syndrome)	Genetic diagnostics

Abbreviations: DRE, drug‐resistant epilepsy; ECG: electrocardiogram; GTCS, generalized tonic–clonic seizures.

* Includes focal to bilateral tonic–clonic seizures.

**TABLE 2:** Patient cohort characteristics.

	Mean (+SD, range) or number (%)
Age	8.8 (4.7, 0–18)
<4 years	69 (14%)
4–6 years	114 (23%)
7–12 years	185 (37%)
>12 years	130 (26%)
Sex	
Female	251 (50%)
Male	244 (49%)
Diverse	3 (1%)
Age at onset of epilepsy	4.4 (4.3, 0–16)
<28 days	33 (7%)
>28 days and <1 year	107 (21%)
>1 year	342 (69%)
Current number of ASM	1.7 (1.3, 0–6)
0	77 (15%)
1	198 (40%)
2	96 (19%)
3+	127 (26%)
Lifetime number of ASM	
0	12 (2%)
1	164 (33%)
2	82 (16%)
3	79 (16%)
4	53 (11%)
5	32 (7%)
6+	76 (15%)
Etiology	
Unknown	207 (42%)
Structural	145 (29%)
Hypoxic–ischemic	51 (35%)
Malformation of cortical development	44 (30%)
Tumor	30 (21%)
Genetic	95 (19%)
Mixed	38 (8%)
Genetic‐structural	31 (82%)
Syndrome	235 (47%)
DEE	140 (60%)
SeLECTS	27 (11%)
Absence epilepsy	17 (7%)
Comorbidity	
Developmental delay	267 (54%)
Cerebral palsy	63 (13%)
Asphyxia	15 (3%)
Seizure semiology	
Focal seizures	228 (46%)
Motor seizures	341 (68%)
Non‐motor seizures	89 (18%)
SUDEP risk factors	
GTCS*	233 (47%)
No seizure freedom (NSF)	192 (39%)
DRE	127 (26%)
Nocturnal seizures	102 (20%)
GTCS* and NSF	80 (16%)
GTCS* and DRE	41 (8%)
Cyanosis	14 (3%)

Abbreviations: ASM, anti‐seizure medication; DEE, developmental epileptic encephalopathy; NSF, no seizure freedom; SeLECTS: self‐limited epilepsy with centro‐temporal spikes.

* Includes focal to bilateral tonic–clonic seizures.

**FIGURE 3:**
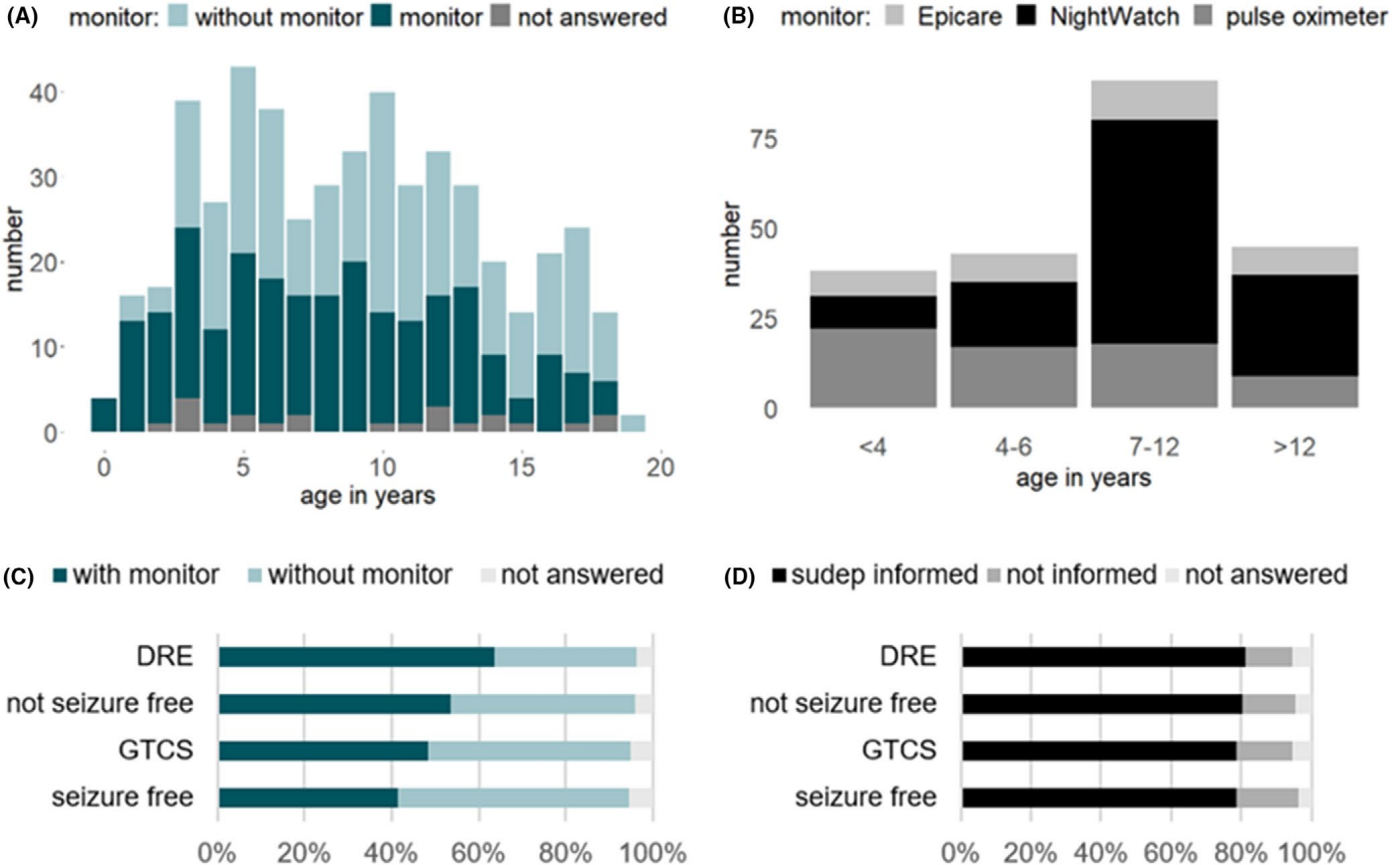
Monitor ownership. (A) According to age. The percentage of those who use a monitor at the age >12 years (35%, *n* = 45) was lower than at the age <12 years (46%, *n* = 86) and <7 years (53%, *n* = 96). (B) Different devices in different age groups. This was primarily due to licensing restrictions. In the <4 years age group pulse oximeter, in the >6 years age group NightWatch® was used most frequently. (C) Monitor ownership depending on SUDEP risk profile. Sixty‐six percent of patients with DRE opted for a nighttime monitoring system. In comparison, 51% of patients who had GTCS* had a monitor; 49% did not. The mere occurrence of the risk factor GTCS* did not significantly influence the decision for or against monitoring. In total, 73% (29/40) of patients at high risk of SUDEP (“non‐seizure‐free,” GTCS* and/or DRE) had a nighttime monitoring system. This means that nearly 30% of patients had no monitoring despite having GTCS*, not being seizure‐free and/or their epilepsy being drug‐resistant. If several risk factors were present at the same time, the decision in favor of a monitor was made more frequently. (D) SUDEP education depending on SUDEP risk profile: In the subgroups DRE, not seizure‐free, GTCS* and seizure‐free in average about 80% were informed about SUDEP. Patients at high risk of SUDEP were informed about SUDEP about the same frequency as patients at low risk. DRE, drug‐resistant epilepsy; GTCS*, generalized tonic–clonic seizures (*including focal to bilateral tonic–clonic seizures). DRE: *n* = 127, not seizure free: *n* = 192, GTCS*: *n* = 233, seizure free: *n* = 268, DRE and not seizure free and GTCS*: *n* = 40, DRE/not seizure free/GTCS*: *n* = 360.

In our cohort, the most frequently used nocturnal monitoring systems were pulse oximeters (29%, *n* = 66) and Nightwatch® (51%, *n* = 117). Other monitoring devices were Epicare 3000®, Epicare free®, Epicare mobile® (7%, *n* = 16), and non‐approved systems (see Figure 3).

A logistic regression was used in this study to investigate whether monitor ownership was significantly related to age, number of ASM, nocturnal seizures, and/or the occurrence of seizure‐associated cyanosis.

**FIGURE 4:**
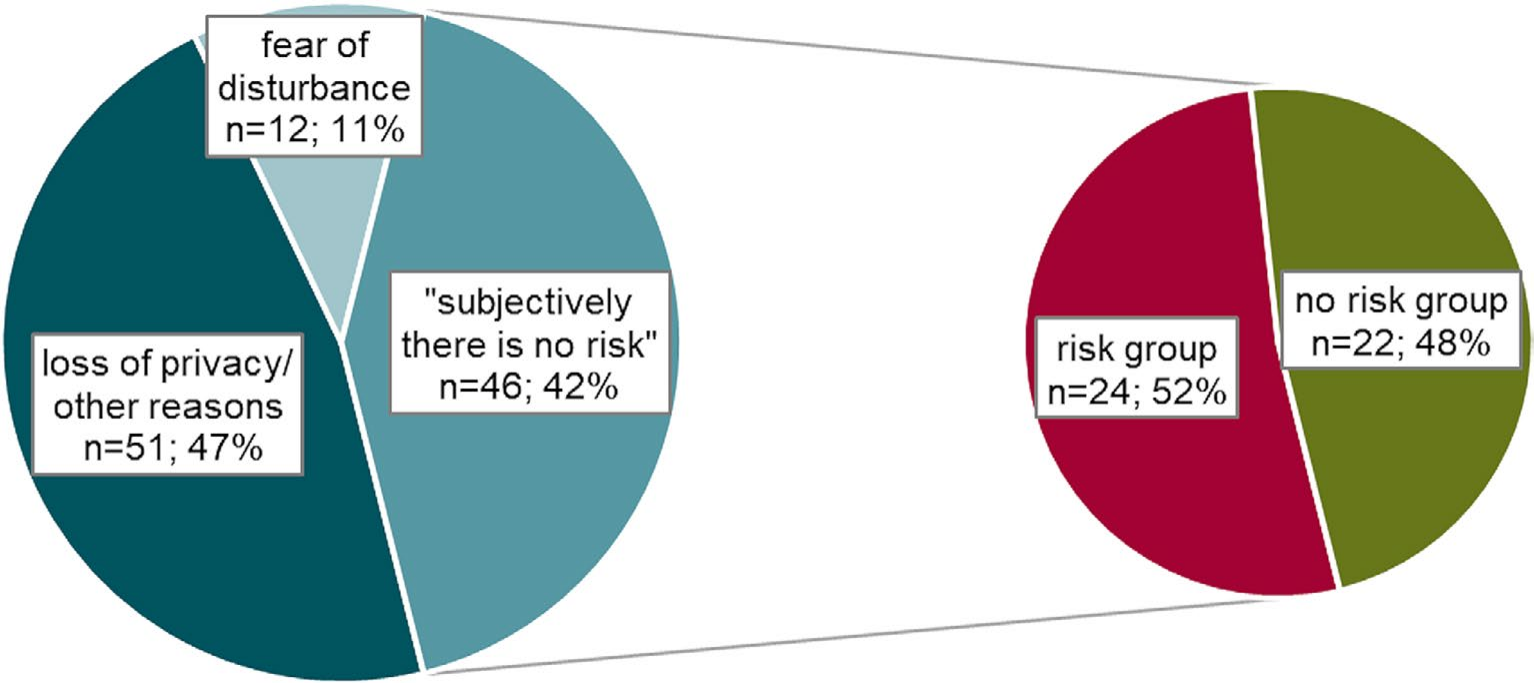
Reasons against monitor ownership. One hundred and nine out of 498 patients (22%) answered when asked why they chose not to have a nighttime monitoring system. When explaining why a nighttime monitoring system was not chosen, a common answer was that “subjectively there is no risk.” Those who stated that “subjectively there is no risk,” 52% (24/46) belong to one of the risk groups for SUDEP: 13/46 were not seizure free, 15/46 have had a GTCS* and/or 7/46 had a drug‐resistant epilepsy (DRE) and 6/46 had a self‐limited centrotemporal epilepsy (SeLECTS).

Age was significantly negatively associated with monitor ownership (odds ratio [OR] = 0.9, 95% confidence interval [CI] = 0.88–0.95, *p* < 0.001), meaning that the likelihood of owning a monitor decreases with age. The number of ASMs was positively associated with monitor ownership (OR = 1.5, 95% CI = 1.27–1.78, *p* < 0.001), indicating an increased likelihood of monitor ownership with an increasing number of medications. Nocturnal seizures showed no association (OR = 1.7, 95% CI = 0.98–2.80, *p* = 0.063). Cyanotic seizures were not significantly associated with monitor ownership (OR = 2.5, 95% CI = 0.70–11.68, *p* = 0.189). Seizure freedom was not directly related to monitor ownership, but it was significantly related to the number of ASMs. This relationship was investigated in a further analysis: The results of Pearson's chi‐square test with Yates' continuity correction showed a significant correlation between seizure freedom and number of medications (*χ* ^2^ = 63.1, *df* = 1, *p* < 0.001). A moderate effect size was observed (Cramér's *V* = 0.36, 95% CI [0.28, 1.00]), but the wide confidence interval suggests considerable uncertainty regarding the true strength of the association (Cohen, 1988). Furthermore, we investigated whether there was a correlation between the occurrence of GTCS (including focal to bilateral tonic–clonic seizures) and monitor ownership. The results of Pearson's chi‐square test with Yates' continuity correction showed no significant correlation between the occurrence of GTCSs and monitor ownership (*χ* ^2^ = 1.26, *df* = 1, *p* = 0.32); the effect size was small (Cramér's *V* = 0.03, 95% CI [0.00, 1.00]; Cohen, 1988). However, the wide confidence interval suggests a high degree of uncertainty in the estimate.

### 3.3 Reasons for monitoring decision

Multiple answers regarding reasons for monitoring were shared by 235 patients. These included a desire to be alerted to cardiovascular or respiratory problems (76%), a desire for better sleep or quieter nights (64%), a desire for information about seizure frequency (56%), a desire for more autonomy or privacy (26%), and in 7% other reasons.

About one third of patients reported that the existing monitoring equipment was not being used. Regardless of which monitor was used, the non‐use rate ranged from 10.5% to 13.3% (Epicare 13.3%, pulse oximeter 12.12%, NightWatch® 11.97%, and others 10.5%). The most common reasons for not using an existing nighttime monitoring system were false alarms (28%), lack of tolerance by the child (25%), feeling of being sick (7%), and failure to detect seizures reliably (7%). Further reasons were given in 32% of cases, including absence of seizures/no perceived risk (*n* = 10), fear of sleep disturbances (*n* = 5), and problems with instructions/application (*n* = 4).

### 3.4 Co‐sleeping

The question about co‐sleeping was answered by 462 of the 498 participants (93%). A total of 39% of guardians stated that they co‐sleep with their child. There was a slight difference in co‐sleeping rates between those with monitoring systems (66%) and those without monitoring systems (51%).

### 3.5 Resuscitation training

At the time of the survey, most caregivers or parents had not taken part in a resuscitation training (77%, *n* = 385), even though this was offered free of charge at our center. This applied regardless of the patient subgroup. Only the parents of patients experiencing seizures with cyanosis completed training in 50% of cases.

### 3.6 Cardiologic diagnostics

At the time of the survey, most patients with epilepsy reported having already undergone a cardiology checkup (58%, *n* = 290). Twenty‐four percent (*n* = 70) stated that they had received a long‐term ECG to assess heart rate variability. Patients at high risk for SUDEP were more likely to undergo cardiologic investigations.

## 4 DISCUSSION

### 4.1 Education about SUDEP

Awareness of SUDEP has increased in recent years,^32^ but the awareness rate remains unsatisfactory.^33^, ^34^, ^35^, ^36^, ^37^, ^38^ Data on SUDEP counseling is primarily available for adult patients with epilepsy. Literature indicates that most doctors do not adequately inform their patients about SUDEP.^39^ One of the first studies to demonstrate this was a 2005 survey by the Association of British Neurologists, in which neurologists reported that only 4.7% of them informed all of their patients with epilepsy about SUDEP, while a maximum of 25.6% of them discussed SUDEP with the majority of their patients.^40^, ^41^ A more recent survey from 2018 showed that only about 31% of 1183 interviewed epilepsy patients had been informed about SUDEP.^38^ In a 2022 worldwide survey of neurologists, 41.5% of doctors still stated that they rarely discuss SUDEP with their patients.^33^ Pediatricians have also reported low levels of SUDEP counseling. Surveys conducted between 2017 and 2018 found that 75.3% of pediatricians were either not aware of the risk of SUDEP^34^ or that only 12% discussed SUDEP with nearly all their patients.^42^ This low counseling rate contrasts with the strong desire of those affected by epilepsy to be informed about SUDEP.^41^, ^43^

In addition to time pressure and stress or unfavorable interpersonal or psychosocial factors, one frequently cited reason for the lack of informing patients or caregivers is that discussing SUDEP evokes discomfort among doctors.^41^, ^44^ This reluctance may stem from a lack of provider knowledge, unfamiliarity with SUDEP, the assumption that another healthcare provider already provided the information, or the belief that the patient is not at risk.^43^, ^44^, ^45^ Providing targeted training for physicians on how and when to discuss SUDEP could be beneficial.^43^, ^44^, ^46^, ^47^ This modifiable factor should be taken into account, especially in view of the fact that SUDEP education can improve patient compliance and potentially reduce risk factors.^43^

Our study provides data on the education rate at the German Center for Epilepsy for Children and Adolescents at Charité. The average education rate among epilepsy patients treated at our center of 92% was achieved primarily through in‐house training of all physicians through lectures and discussions, providing a SUDEP information one‐pager to facilitate discussions with patients and caregivers, and including information on the SUDEP awareness and monitoring status in the formal patient letters. This result is comparable to the impact of the SUDEP and Seizure Safety Checklist, which has been used in adult patients in the United Kingdom since 2015 as a catalyst for SUDEP education.^29^

The introduction of standardized questioning about SUDEP awareness status using a parent questionnaire not only led to the recording of the current situation and the identification of information gaps, but also to an increase in the awareness rate through the subsequent provision of information in cases where awareness was lacking. Notably, there were no significant differences in awareness rates between subgroups, including those at high risk for SUDEP. This indicates that patients with high SUDEP risk factors were not informed more frequently than those without risk factors. A considerable proportion of patients first received education about SUDEP months to years after their epilepsy diagnosis—only when they first presented at our center—despite recommendations for early SUDEP education following epilepsy diagnosis.^43^, ^44^ This recommendation also corresponds to the preference of around 67% of parents who would like to be informed at the time of diagnosis.^39^

### 4.2 Monitor ownership

While the SUDEP awareness rate does not differ significantly between the various SUDEP risk subgroups (see Figure 3 and Table 2), our study suggests different behaviors across age subgroups and SUDEP risk groups regarding the acquisition of nighttime monitoring systems or seizure detecting devices. In particular, a younger age and the number of ASM appeared to have a positive influence on the decision to acquire a detection device. The younger the patients, the more frequently their parents or caregivers opted for a monitor, despite the fact that risk factors like GTCS, focal to generalized tonic–clonic seizures, DRE, and lack of seizure‐freedom did not depend on age. Specifically, 69% of parents or guardians of children under 4 years of age opted for monitoring, whereas only 35% of caregivers of patients over 12 years opted for a detection device. This observation is congruent with a study by Chiang et al. and Engelgeer et al.,^48^, ^49^ while Borusiak found no significant effect of age on monitor ownership in his survey of 153 patients under the age of 18 years.^27^ Further studies are needed to explain why caretakers of children under 4 years of age are most likely to opt for nighttime monitoring.

There was a high level of interest among caregivers of children under the age of 4 years with epilepsy in acquiring a monitoring device. However, at the time of the study, only two devices were approved for nighttime monitoring in Germany: (i) A pulse oximeter, which was not suitable for detecting seizures but only monitored heart rate and oxygen saturation. (ii) The mattress sensor Epicare 3000®, a device approved for seizure detection from the age of 6 months. Thus, only a limited range of products was available to meet the needs of this age group, leaving a gap in monitoring options for children younger than 6 months. At the same time, the SUDEP risk does not decrease with age, and solutions should also be developed for older age groups to increase the willingness to use nighttime monitoring despite the growing need for autonomy.

The inadequate supply situation in Germany serves as an example for other countries as well. Other countries also report supply shortages and, in particular, a lack of reimbursement systems.^50^ Given the complexity and diversity of existing devices, a detailed and individual consultation is often essential,^51^ and national guidelines would be useful.^52^ The ILAE also advocates the use of nocturnal monitoring systems, especially for high‐risk patients, after seizure control as the primary goal to reduce the SUDEP risk (expansion of all treatment options, improvement of compliance, and lifestyle adjustments).

Our study indicates that a higher number of ASMs is associated with a greater proportion of patients, parents, or caregivers opting for a seizure detection device. According to our study, lack of seizure freedom also had a positive influence on the decision to use nighttime monitoring. This observation is consistent with the findings of van Westrhenen in the Netherlands.^53^ Given the increased SUDEP risk in patients with DRE,^12^ nighttime monitoring—such as with NightWatch®—may aid in seizure detection, alert parents or caregivers, and thereby reduce anxiety and worry.^49^

There was a discrepancy between different risk constellations or risk groups. While DRE was obviously identified more often as a risk factor, the main risk factor for SUDEP—GTCS and focal to generalized tonic–clonic seizures (GTCS*)—was not always identified as such by their caregivers. Thus, the presence of the primary risk factor GTCS* did not appear to influence risk‐related behavior. While 66% of patients with DRE or their caregivers opted for a nighttime monitoring system, the probability of opting for a monitoring system with GTCS* was around 50%. Specifically, among patients who have ever had a GTCS*, 51% had a monitor; 49% did not. Borusiak's study also showed that the presence of the main risk factor for SUDEP did not influence risk behavior in terms of monitor ownership.^27^ This underscores a greater need for education, particularly for patients with GTCS* in their patient's history.

There is no evidence that nighttime monitoring prevents SUDEP risk. Existing evidence is observational or based on plausibility, not on ethically difficult randomized controlled trials.^26^, ^27^, ^28^, ^50^ Future research should use ethically appropriate designs (e.g., observational studies, stepped‐wedge designs) to clarify potential benefits. Monitoring may intrude on privacy and autonomy, so future studies must assess usability and whether a general recommendation for nighttime monitoring is justified. Still, seizure detection devices have the potential to reduce the uncertainty associated with epilepsy: In surveys, parents reported that using a monitor helped to reduce seizure‐related anxiety by 80%, with no significant difference in quality of life between users and non‐users.^30^, ^48^

### 4.3 Reasons against monitoring

The individual decisions of patients and/or their caregivers against monitoring were based on multiple reasons (Figure 4). Critically, the decisions regarding “the absence of danger” were not always based on correct assumptions or misjudgments. In our study, a significant proportion of respondents (42%, 46/109) stated that patients/caregivers had decided against monitoring because “in their opinion there is no danger.” However, more than half (52%, 24/46) of these respondents belonged to at least one of the following high SUDEP risk groups: GTCS*, DRE, lack of seizure‐freedom, nocturnal seizures, or seizures with cyanosis. This suggests a lack of awareness regarding their high‐risk status, partly despite receiving information during counseling. This finding is in line with a study conducted in adults using seizure detection devices,^54^ which highlighted that clinical risk profiles do not always correspond with risk management decisions or willingness to use seizure detection devices.^54^ This underscores the need for improved SUDEP education, including the risk factors.^44^ It is also striking that only 90 patients answered this group of questions. Most patients without a monitor (63%, 156/246) did not state a reason, suggesting limited engagement with the topic. Potential barriers include low trust in technology,^55^ physicians underestimating patient risk,^43^ and financial constraints,^50^ especially where reimbursement is lacking. Additional socioeconomic and psychosocial factors—such as living conditions, privacy concerns, sleep disruption, perceived overprotection, loss of autonomy, and fear of stigmatization—also shape decisions. Overall, choices for or against monitoring reflect a balance between safety, quality of life, and social reality. This study does not claim that all individuals with GTCS* should use nighttime monitoring; further evidence is needed. However, the findings show limited patient awareness of GTCS* as the main SUDEP risk factor. Improved risk‐specific education is essential to support informed decisions about preventive strategies, including the potential role of nocturnal monitoring in SUDEP prevention.

Knowing that unobserved sleep is a risk factor for SUDEP, one would expect that patients/caregivers who decide against using a monitoring device would be more inclined to co‐sleep, as suggested by Leeuwen.^54^ However, this was not the case in our cohort. In fact, patients/caregivers who used a monitor were more likely to co‐sleep than those without a monitor. This suggests that individuals who choose not to monitor also choose not to co‐sleep because they perceived the overall risk of SUDEP to be low. And it might also indicate that the patients/caregivers do not fully trust seizure detection devices, as mentioned earlier.^55^

The number one reason in our survey for not using an existing monitor was false alarms. This highlights the need for the development of more reliable seizure detection devices, as also emphasized by a recent multicenter study in the Netherlands and the United States of America.^30^, ^56^ Although NightWatch® was the most commonly used device in our study and that of others^27^ and has a high sensitivity (median 100%, range 46%–100%),^30^ many families in our survey still reported experiencing false alarms. Another reason for discontinuing the use of an existing monitor was rejection by the child. The most common comorbidities in patients with epilepsy were developmental disorders. Cognitive impairments can contribute to a lack of acceptance of such devices that need to be attached to the body. Additionally, limited communication skills, such as in speech‐impaired patients, make it more difficult to explain the need for cooperation. This further highlights the need for seizure detection devices that are comfortable to wear or barely noticeable to improve acceptance. Investment in the development of reliable seizure detection devices that are comfortable to wear is therefore desirable.

### 4.4 Cardiologic diagnostics

In the SUDEP cases described by Ryvlin (MORTEMUS), arrhythmia did not appear to be the primary event.^10^ But recent studies and, above all, molecular genetic findings suggest that cardiac factors appear to play a greater role in the development of SUDEP.^57^ Channelopathies affecting both the brain and the heart are gaining recognition and could help explain the links between epileptic and arrhythmogenic/cardiac syndromes.^58^ For example, gene variants that affect sodium and potassium channel function—already known in patients with cardiac arrhythmias or who have died of sudden cardiac death—are now suspected to play a role in SUDEP (e.g., potassium channel gene *KCNH2* variants).^59^ However, individuals with a genetic predisposition to sudden cardiac death may be more vulnerable in such contexts.^60^ The individual genetic background may play an important role. Also, anti‐seizure medication (ASM) such as sodium channel blockers can trigger cardiac arrhythmia.^61^ In addition, “The Epileptic Heart Syndrome” may play a role in cardiac fibrosis associated with SUDEP.^62^ For example, it has been reported that cardiac fibrosis was found more frequently in patients with sudden unexpected death in epilepsy (SUDEP) than in control samples.^63^

We therefore recommend generously that all children with chronic epilepsy or uncertainties regarding the diagnosis undergo an ECG and, if abnormalities are found, be referred to a cardiologist, especially patients at high risk for SUDEP (GTCS*, nocturnal seizures, and DRE). Further research is needed to establish whether neurology/neuropediatrics should adopt a recommendation similar to that of cardiology societies, namely systematic molecular genetic testing combined with precise family history.^64^

## 5 CLINICAL RELEVANCE

Our findings underscore the urgent need for standardized SUDEP awareness education, such as the structured approach at our center including in‐house training of physicians and of parents or caregivers, the use of a standardized questionnaire and the documentation of SUDEP risk and prevention measure counseling status. Despite existing knowledge, SUDEP risk factors are frequently underestimated, warranting intensified efforts to bridge this critical information gap.

Additionally, age‐specific needs must be systematically addressed, ensuring that all patient groups receive targeted risk education and support. Given the vulnerability of young children, we emphasize the necessity of developing highly reliable seizure detection devices that are comfortable to wear, particularly for children under 4 years of age.

A multidisciplinary approach involving clinicians, researchers, policymakers, patients, and their families is needed to effectively implement these measures, enhance risk management strategies, and strengthen SUDEP prevention efforts.

## 6 LIMITATIONS

Direct evidence for a significant reduction in the risk of SUDEP through nocturnal monitoring is still limited; existing studies suggest that systems such as NightWatch® may be a valuable addition to the monitoring of nocturnal seizures. By detecting seizures early, they enable faster intervention, which could potentially reduce the risk of SUDEP.^65^ Another limitation of this study lies in the dual role of the questionnaire, which functioned simultaneously as an intervention tool and as an outcome measure. Therefore, the observed improvements in educational outcomes are inherently linked to the process of completing the survey and receiving feedback. This overlap may have led to an artificial inflation of the measured effect. To mitigate this limitation in future research, the intervention and outcome assessment should be separated from each other. Due to the lack of longitudinal follow‐up in this case, the educational effect of the 25% increase in “SUDEP awareness” may have been overestimated. In a future study, the sustainability of this “awareness campaign” could be supplemented by measuring behavioral changes (compliance, reporting of seizures, and emergency preparedness).

## FUNDING INFORMATION

The study was supported by the Einstein Stiftung Fellowship through the Günter Endres Fond, the Sonnenfeld‐Stiftung, the Federal Ministry of Education and Research (Bundesministerium für Bildung und Forschung, BMBF) as part of the German Center for Child and Adolescent Health (DZKJ, 01GL2401A), and the Charité—Universitätsmedizin Berlin. None of the authors has any conflict of interest to disclose.

## CONFLICT OF INTEREST STATEMENT

None of the authors has any conflict of interest to disclose.

## ETHICS STATEMENT

We confirm that we have read the Journal's position on issues involved in ethical publication and affirm that this report is consistent with those guidelines. The study was conducted in accordance with the Declaration of Helsinki and approved by the Institutional Review Board (or Ethics Committee) of Charité—Universitätsmedizin Berlin (EA2/112/24).

## Supporting information


Data S1:

## Data Availability

The data are available upon reasonable request to the corresponding author. However, due to data protection or ethical restrictions, not all data are available.
