# Supplementary material for: Sudden unexpected death in epilepsy (SUDEP): Risk management of pediatric patients with epilepsy
Source: Epilepsia Open. 2026 Jan 20;11(2):422–34. doi: 10.1002/epi4.70214 (PMC13051995; doi:10.1002/epi4.70214)
Supplement: Supplementary file 1 — Data S1: [file EPI4-11-422-s001.docx]

**Supplemental Figures**


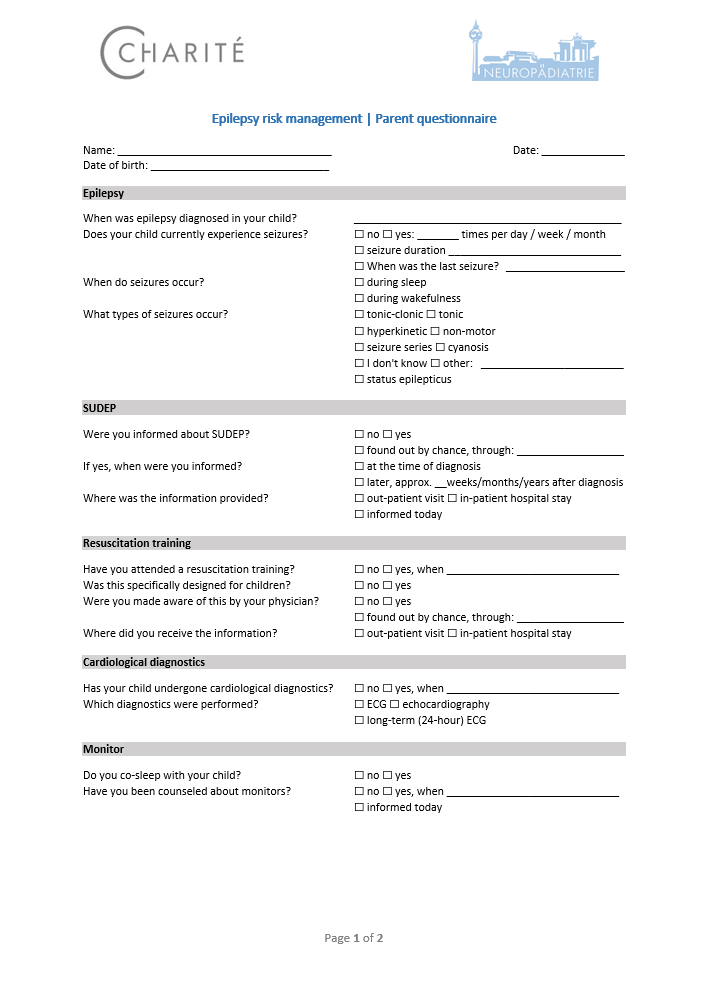


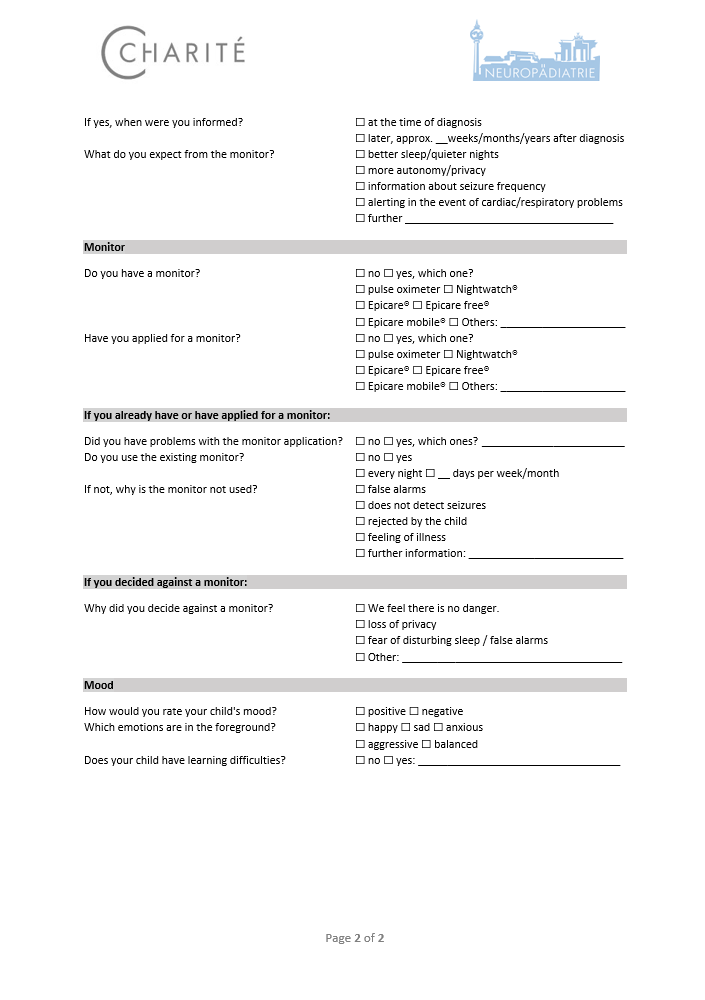


**Supplemental Figure 1.** Survey questionnaire to record knowledge of preventive measures regarding SUDEP. Survey questionnaire in English.


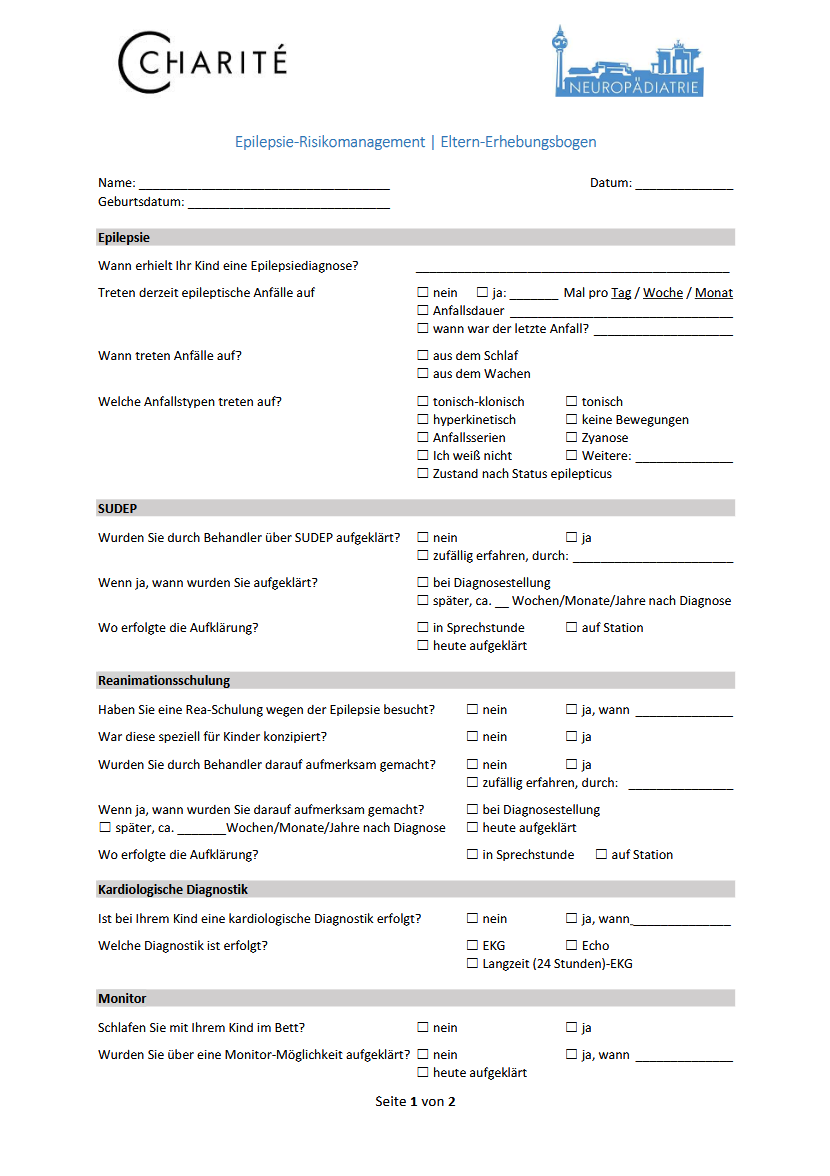


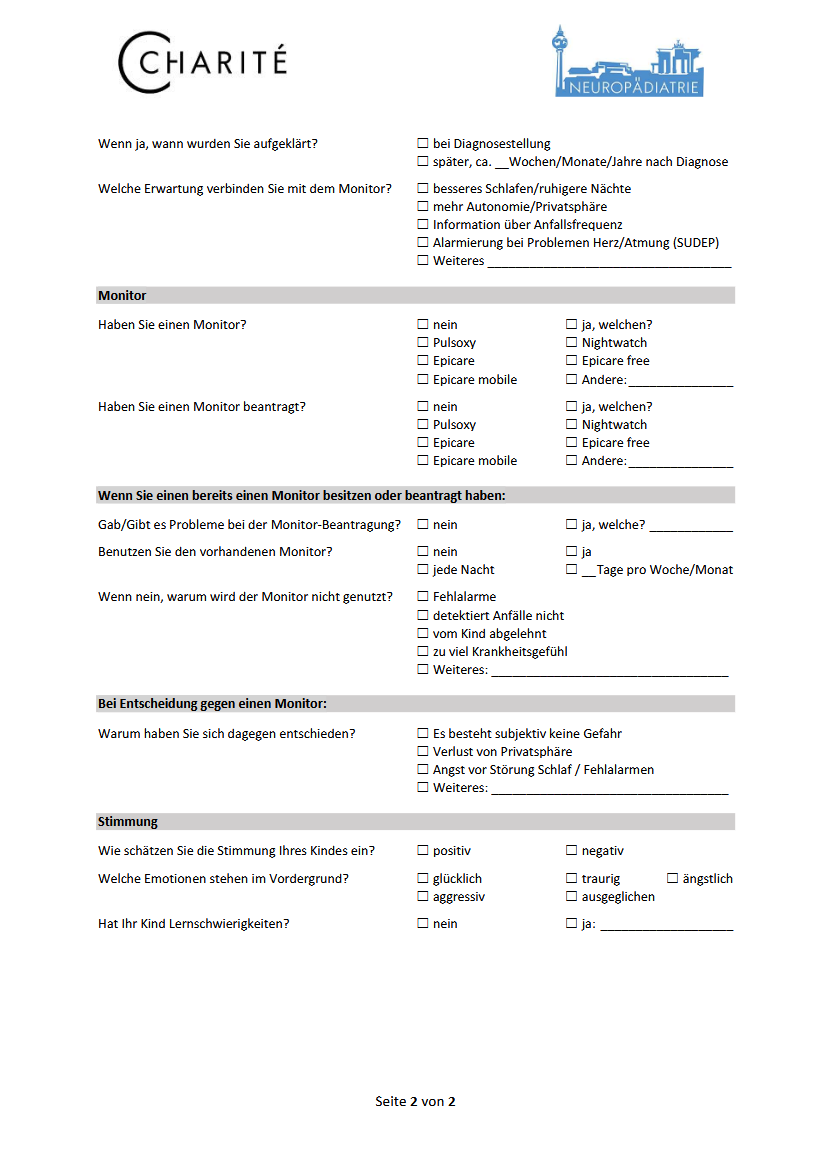


**Supplemental Figure 2.** Survey questionnaire to record knowledge of preventive measures regarding SUDEP. Survey questionnaire in German.
